# Complete Genome Sequence of *Streptomyces* Siphophage Sitrop

**DOI:** 10.1128/MRA.01337-20

**Published:** 2021-01-07

**Authors:** Victor Portillo, Haydee Diaz, James Clark, Isla Hernandez, Mei Liu, Ben Burrowes

**Affiliations:** a Department of Biochemistry and Biophysics, Texas A&M University, College Station, Texas, USA; b Center for Phage Technology, Texas A&M University, College Station, Texas, USA; DOE Joint Genome Institute

## Abstract

*Streptomyces* sp. strain Mg1 is a Gram-positive soil bacterium capable of causing cell lysis and degradation of Bacillus subtilis colonies. Here, we report the 48,481-bp genome of *Streptomyces* sp. Mg1 siphophage Sitrop. With 77 predicted protein-coding genes and one tRNA, Sitrop shares 77% nucleotide sequence identity with the *Streptomyces* phage Verse.

## Abstract (precis)

*Streptomyces* sp. strain Mg1 is a Gram-positive soil bacterium capable of causing cell lysis and degradation of Bacillus subtilis colonies. Here, we report the 48,481-bp genome of *Streptomyces* sp. Mg1 siphophage Sitrop. With 77 predicted protein-coding genes and one tRNA, Sitrop shares 77% nucleotide sequence identity with the *Streptomyces* virus Verse.

## ANNOUNCEMENT

*Streptomyces* sp. strain Mg1 is a Gram-positive, saprotrophic bacterium predominantly found in soil (1, 2). This filamentous organism is known to produce the antibiotic chalcomycin A, which plays a role in competition with and inhibition of Bacillus subtilis (2). Streptomycetes produce several useful secondary metabolites, including approximately 80% of today’s antibiotics (3). Studying the genomes of phages of industrially important bacterial species, such as *Streptomyces* sp. Mg1 siphophage Sitrop, may be useful for improving bioproduction technologies.

Bacteriophage Sitrop was isolated in February 2019 from a soil sample taken from Lincoln, Nebraska. Sitrop was plaque purified as described elsewhere (4) using *Streptomyces* sp. Mg1 as its host and cultured at 30°C on nutrient broth or agar supplemented with 10 mM MgCl_2_, 8 mM Ca(NO_3_)_2_, and 0.5% glucose. Sitrop was found to be chloroform sensitive. DNA was purified using a modified Wizard DNA clean-up kit (Promega) protocol (5) and prepared as Illumina TruSeq libraries. Sequencing was performed on an Illumina iSeq 100 instrument with paired-end 300-bp reads using a TruSeq Nano DNA kit. Quality control of the 254,194 resulting sequence reads was done using FastQC (http://www.bioinformatics.babraham.ac.uk/projects/fastqc) and trimmed manually with FastX 0.0.14 (http://hannonlab.cshl.edu/fastx_toolkit/download.html). The genome was assembled into a single contig at 109.8-fold coverage using SPAdes v3.5.0 (6) and closed by PCR and Sanger sequencing of the resulting product using primers GGACGTTGAACTTGTTGAGGA (forward) and GTCCTCCAGGTGTGAAGAAG (reverse). Structural annotations of protein-coding genes were initially predicted by GLIMMER v3 (7) and MetaGeneAnnotator v1.0 (8), while tRNAs were found using ARAGORN v2.36 (9). Conserved domains, sequence similarity, and transmembrane domains were found using InterProScan v5.33 (10), BLAST v2.9.0 (11), and TMHMM v2.0 (12), respectively, to predict gene function. BLAST similarity searches used a 0.001 maximum expectation value cutoff against the NCBI nonredundant, Swiss-Prot, and TrEMBL databases (13) (accessed 8 April 2020). The DNA sequence similarity of the entire genome was calculated with progressiveMauve v2.4 (14). Annotation tools used in Galaxy and Web Apollo are hosted at https://cpt.tamu.edu/galaxy-pub (15) by the Center for Phage Technology. Phage virion morphology was visualized as 2% (wt/vol) uranyl acetate negatively stained samples by transmission electron microscopy at the Texas A&M Microscopy and Imaging Center and determined to be a siphovirus (data not shown). All tools were run with default parameters unless otherwise specified.

Sitrop is a siphophage with a genome of 48,481 bp and a G+C content of 65.6%, compared to 72.17% of its host. The genome was composed of 1 tRNA gene and 77 predicted protein-coding genes, 41 being assigned putative functions, with a total coding density of 89.7%. Sitrop is most closely related to *Streptomyces* phage Verse (GenBank accession number KT186229.1), having 76.93% nucleotide identity and 67 shared proteins (16). Sitrop also shared close similarity to other *Streptomyces* phages within the *Camvirus* genus. Most of the protein-coding genes predicted to be tail proteins appear to be novel, sharing significant amino acid sequence similarity only with *Streptomyces* phages Alsaber (MG298964.1) and Saftant (MN204498.1), two novel phages with close similarity to Sitrop overall. There were four genes predicted to be involved in lysis, including an endolysin endopeptidase, a holin, and a separated two-component spanin complex gene (17).

### Data availability.

The genome of Sitrop is available in GenBank under accession number MT701598.1. The associated BioProject, SRA, and BioSample accession numbers are PRJNA222858, SRR11558341, and SAMN14609631, respectively.
